# Understanding the Dengue Viruses and Progress towards Their Control

**DOI:** 10.1155/2013/690835

**Published:** 2013-07-09

**Authors:** Rosmari Rodriguez-Roche, Ernest A. Gould

**Affiliations:** ^1^“Pedro Kouri” Tropical Medicine Institute, WHO/PAHO Collaborating Centre for the Study of Dengue and Its Vector, Autopista Novia del Mediodía, Km 6 1/2, La Lisa, P.O. Box 601, Marianao 13, 11300 Havana, Cuba; ^2^UMR190, Emergence des Pathologies Virales, Aix-Marseille Universite, Institut de Recherche pour le Développement, Ecole des Hautes Etudes en Santé Publique, Unité des Virus Emergents, Faculté de Médecine de Marseille, 27 Boulevard Jean Moulin, 13005 Marseille cedex 05, France; ^3^CEH Wallingford, Maclean Building, Benson Lane, Crowmarsh Gifford, Oxfordshire OX10 8BB, UK

## Abstract

Traditionally, the four dengue virus serotypes have been associated with fever, rash, and the more severe forms, haemorrhagic fever and shock syndrome. As our knowledge as well as understanding of these viruses increases, we now recognise not only that they are causing increasing numbers of human infections but also that they may cause neurological and other clinical complications, with sequelae or fatal consequences. In this review we attempt to highlight some of these features in the context of dengue virus pathogenesis. We also examine some of the efforts currently underway to control this “scourge” of the tropical and subtropical world.

## 1. Introduction

Dengue fever is a mosquito-borne virus disease of humans. In terms of numbers of individuals infected, it is by far the most devastating of all the recognised arthropod-transmitted virus diseases. It is estimated that more than 3 billion humans live in dengue endemic regions of the world, and currently, more than 50 million infections occur annually with at least 500,000 individuals requiring hospitalisation [1]. Of these, tens of thousands have a high risk of developing haemorrhagic disease, potentially with fatal consequences depending to a large extent on the quality of the available medical services. 

The dengue viruses are positive stranded RNA viruses in the genus *Flavivirus*, family *Flaviviridae *[2]. There are four distinct dengue virus (DENV) serotypes that share antigenic relationships (DENV-1, DENV-2, DENV-3, and DENV-4), and although infection with one serotype confers lifelong protection against that serotype, it does not necessarily protect against a secondary infection with a heterologous serotype. Indeed, nonprotective but cross-reactive antibodies may enhance disease severity [3]. Currently, there are no effective vaccines or antiviral drugs against these viruses. This problem is being addressed as a matter of urgency as failure to develop effective DENV control strategies will inevitably result in a further increase in the number of infected humans, as predicted more than a decade ago [4]. This problem is also exacerbated by the continuing dispersal of these viruses to new geographic regions. 

This review therefore focuses on our current understanding of dengue virus pathology, epidemiology, pathogenesis, evolution, biogeography, and disease control. 

## 2. Dengue Fever/Haemorrhagic Fever/Shock Syndrome

### 2.1. Clinical Picture

In most cases asymptomatic or relatively mild disease follows infection with dengue virus. However, to take into account the increasing number of clinical cases, the World Health Organization (WHO) produced guidelines [5] in which they identified the clinical pictures resulting from infection with dengue virus. The first, known as dengue fever (DF), is characterised by an abrupt onset of fever accompanied by frontal headache and retroorbital pain, followed by a variety of possible clinical symptoms such as myalgia, arthralgia, vomiting, and weakness. A generalised maculopapular rash appears one or two days after fever defervescence. Minor haemorrhagic manifestations such as petechiae may be observed in some patients. DF is generally self-limiting and rarely fatal. Most patients recover without complications around ten days after the onset of illness. 

The second clinical picture, dengue haemorrhagic fever (DHF), is a more severe form of the disease and occurs in up to 5% of dengue cases. It is initially characterized by the same variety of clinical symptoms as are seen in DF. The critical period in DHF starts at the moment of defervescence but haemorrhagic manifestations may occur 24 hours earlier. A positive tourniquet test indicates that the patient has increased capillary fragility. Petechiae, bleeding at venepuncture sites, epistaxis, gum bleeding, and haematemesis may also be observed. High fever, haemorrhagic manifestations, thrombocytopenia (platelet count 100 000/mm^3^ or less), and haemoconcentration (>20% difference) characterize DHF. Plasma leakage is the most significant pathophysiological event in determining the severity of the disease. Signs of circulatory failure such as irritability, cold clammy extremities, flushed face, and restlessness may be observed. This crisis usually persists for 24–36 hours. With appropriate supportive medicine and carefully monitored intravenous isotonic crystalloid therapy, to ensure adequate fluid replacement, most patients recover. However, during this critical period, it is essential to look for characteristic warning signs of worse to come. Patients progressing to shock (dengue shock syndrome—DSS) show intense abdominal pain or tenderness, persistent vomiting, weak pulse, and hypotension. If increased vascular permeability progresses to vascular collapse the outcome is usually fatal as a result of irreversible DSS. In addition to DF, DHF, and DSS, it is now recognised that other clinical manifestations can be associated with infection by dengue virus, for example, encephalitis, myocarditis, hepatitis, cholecystitis, myelitis, and acute colitis [6–11]. 

Despite the rigour of the DF/DHF/DSS classification and its intrinsic worth in clinical case management, in recent years, a growing number of clinicians and authors have argued that the 1997 WHO scheme for dengue clinical classification should be reassessed [12–14] because it distinguishes strictly between DF, DHF, and DSS, whereas it is now recognised that the point of transition between DF and DHF is not easily defined. The requirements for the WHO definition of DHF (fever, haemorrhage, thrombocytopenia, and signs of plasma leakage) are not always satisfied; severe thrombocytopenia may be observed in uncomplicated as well as severe cases associated with “unusual manifestations” and therefore may not be consistent with the DHF/DSS classification [15]. A WHO/TDR-supported prospective clinical multicentre study across dengue-endemic regions was established to collect and coordinate specific criteria for classifying clinical cases into levels of severity [16]. The study confirmed that, by using a set of clinical and/or laboratory parameters, one sees a clear-cut difference between patients with severe dengue fever and those with nonsevere dengue fever. However, for practical reasons it was considered necessary to split the large group of patients with nonsevere dengue into two subgroups: patients with warning signs (abdominal pain or tenderness, persistent vomiting, clinical fluid accumulation, mucosal bleeding, lethargy, restlessness, liver enlargement >2 cm, and increase in hematocrit concurrent with rapid decrease in platelet count) and those without these warning signs. On the other hand, the criteria for severe dengue fever include extensive plasma leakage, severe bleeding, or severe organ impairment. 

The third and most recent edition of the WHO/TDR dengue guidelines for diagnosis, treatment, prevention, and control includes a new clinical classification [17]. This publication serves as an authoritative reference source for health workers and researchers. These new guidelines provide a revised case classification which is intended to facilitate effective triage and patient management and collection of improved comparative surveillance data [18]. However, due to the recommendation that cases of dengue fever with warning signs and also cases of severe dengue fever should be admitted to hospital, there is concern that this could result in overadmission of patients to hospitals during epidemics, inevitably reducing the efficiency of patient triage and adversely affecting the quality of clinical case management [19, 20]. Furthermore there is additional concern that the WHO/TDR classification may impact significantly on dengue pathogenesis research since it requires the identification and study of distinct dengue syndromes. Because the 2009 WHO case definitions do not require laboratory tests for the diagnosis of severe dengue, it is considered that retrospective identification of patients with clinically significant vascular permeability, from data on hospital charts, may be difficult if not impossible [21]. The previous discussion highlights the difficulties of designing a totally acceptable classification scheme for dengue pathogenesis.

### 2.2. History of DF and DHF

Clinically diagnosed DF was widespread during the 18th and 19th centuries in North and South America, the Caribbean Basin, Asia, and Australia. In the Americas, this was largely due to the repeated introduction from Africa of *Stegomyia (St.) aegypti *(formerly *Aedes aegypti*) [22–24]. Moreover, together with yellow fever virus (YFV), DENV-infected humans and mosquitoes were introduced via the slave ships and other commercial vessels that crossed the Atlantic Ocean from Africa during the past five or more centuries [25–31]. 

It is also important to note that disease clinically compatible with the more severe and often fatal syndromes, DHF and DSS, was sporadically reported from 1780 onwards [32] although it is not clear if the more severe cases were confined to individuals of European descent. 

DF thus became endemic in Latin America and the Caribbean region, periodically causing epidemics. At the same time, YFV was also causing epidemics in South America, prompting the Pan American Health Organisation (PAHO) to introduce a mosquito-eradication programme which lasted from 1946 until the late 1970s. Since both DENV and YFV are transmitted to humans via *St. aegypti* this eradication campaign in South America also resulted in a lower incidence of DF in South America. Thus, DF was confined mainly to the Caribbean basin [33, 34]. Subsequently, the gradual decline of mosquito control measures and increasing introduction and dispersal of mosquitoes via transportation for commercial and military purposes led to the reemergence of dengue as a major health problem during the mid and later parts of the 20th Century. The incidence of dengue fever increased dramatically in Southeast Asia during World War II and continued to intensify with increased geographic spread of the viruses and the principal mosquito vector, *St. aegypti*. In addition to the major influence of increased shipping and air-traffic globally, other major factors for the reemergence of dengue fever include ecological and demographic changes in the tropical zones [2, 34–39]. 

During the 1980s and 1990s, rapidly expanding populations of *St. aegypti* in Brazil resulted in successive epidemics due to DENV-1, DENV-2, and DENV-3. In Brazil, these infections presented mostly as DF, with surprisingly few cases of DHF. This contrasts with Asia where the proportion of DHF cases was significantly higher during DF epidemics. These differences have been partly attributed to the widespread presence of dengue virus resistance genes in Latin Americans with African ancestry [33, 40, 41]. The differences may also be partly explained by the high levels of antibody against the American DENV-2 genotype and antigenically cross-reactive DENV-1, both of which had been endemic in Latin America for many years. 

Today, all four DENV serotypes circulate in Africa, South and Southeast Asia, the Western Pacific region, the Caribbean basin, and Central and South America [39, 42–44]. Frequent introductions into the Southern states of North America are also regularly recorded although to date they have not resulted in epidemic outbreaks in the USA; DF has the potential to become reestablished as an endemic disease in this country. In fact, sustained transmission of dengue has occurred in Florida during recent years. Conditions exist that could facilitate sustained dengue transmission, including environmental factors, competent mosquito vectors, limited vector and dengue surveillance, increased domestic outdoor daytime activities in warmer months, and low public awareness of the disease [45]. Indeed, dengue continues to spread more widely as demonstrated in 2010 by the first recorded cases of autochthonous dengue fever in southern France [46] and Croatia [47].

Many countries in the tropics and subtropical regions show cocirculation of at least two DENV serotypes [36], and increasingly, cocirculation of all 4 serotypes is being recorded in individual countries. Taken together with the ecological and demographic changes, this partly explains why the pattern of epidemics is gradually increasing from a frequency of outbreaks every 3–5 years to approximately every 2 years [48]. Additional explanations for this increased incidence include the possibility that more highly pathogenic strains of DENV are also emerging [44, 49–51]. Greater awareness of this disease, as the result of more extensive monitoring, is also impacting on our understanding of and the apparent increased periodicity of dengue virus epidemiology. 

Comparison of disease incidence in Asia and Latin America reveals a distinct difference in the age distribution of DF and DHF. In Asia, hospitalizations principally involve children, whereas in the Americas, they tend to involve a greater proportion of adults [33]. The reasons for this apparent difference have not been adequately defined. However, to complicate this issue, a recent epidemic in the State of Rio de Janeiro revealed that the incidence of DHF in children was significantly higher than in previous epidemics in Brazil [52, 53]. 

### 2.3. Risk Factors Associated with the Development of Severe Dengue

The principal vector associated with all 4 DENV serotypes is the African mosquito *St. aegypti *an urban-dwelling anthropophilic mosquito. However, *St. albopicta *the Asian “Tiger” mosquito is also competent to reproduce and transmit DENV between humans. In contrast with *St. aegypti, St. albopicta *is peridomestic, with a preference for the rural environment. In some parts of Asia and Africa, *St. albopicta *has displaced *St. aegypti *[54, 55]. A possible scenario of this changing pattern of mosquito distribution and the continuing dispersal of *St. albopicta* is that the dengue viruses will disperse even more widely, gradually establishing in the warmer regions of the temperate zones, including Europe [46, 47, 56, 57], the southern regions of North America [58], and more northern regions of Asia [59]. 

The pathogenetic basis for DHF has been a subject of study for decades, and whilst significant progress has been made in understanding the most important risk factors involved, the precise biochemical and immunological pathways have not yet been defined [60–62]. Amongst the several possibilities that have been identified, there is compelling evidence that secondary infection with a heterologous DENV serotype, or primary infection in infants born to dengue-immune mothers, is an important individual risk factor for DHF/DSS [63–70]. During secondary infection with a different serotype, the presence of low levels of heterotypic neutralizing antibodies may reduce disease severity. Alternatively, in the absence of such neutralizing antibodies, heterotypic cross-reactive antibodies may form complexes with the virus and the Fc-receptors on these complexed antibodies may attach to mononuclear phagocytes, thus enhancing the efficiency of infection and thereby increasing the number of infected mononuclear phagocytes [71–74]. This phenomenon is known as antibody dependent enhancement (ADE) [75]. Humans infected with one serotype maintain a life-long protective immunity to infection by the homologous virus, but protective immunity to infection with heterologous serotypes is relatively short-lived [76]. The precise mechanism by which DENV replication is amplified in the infected cells remains unclear. One possibility is that there is a relationship between DENV-ADE infection, suppression of nitric oxide during the innate immune response, and the corresponding cytokine-expression pattern in THP-1 cells [77]. Recent evidence suggests that viral susceptibility or resistance to nitric oxide may be regulated by the viral NS5 protein [78].

 It has also been argued that strain differences in virulence may contribute to disease severity [51, 79–84]. However, the fact that severe dengue disease is identified most consistently following secondary dengue infections supports the view that virulence must be defined in a two-infection context [39]. Host risk factors such as gender, ethnicity, the presence of chronic disease (bronchial asthma, diabetes mellitus, and sickle cell anaemia) [85–87] and also the genetic characteristics of the individual are also likely predisposing factors for severe illness. Human leukocyte antigen (HLA), Fc*γ*R, tumor necrosis factor- (TNF-) *α*, and dendritic cell-specific intracellular adhesion molecule-3-grabbing nonintegrin (DC-SIGN), among other genes, have been associated with the pathogenesis of dengue [41, 66, 86, 88–93]. In addition, T-cell mediated immune mechanisms, involving skewed cytokine responses resulting in plasma leakage, are also risk factors for DHF [62]. It has been proposed that an inappropriate immune response to the secondary virus infection, that in turn induces reactivation of cross-memory T cells specific for the first rather than the secondary DENV infection, results in delayed viral clearance [94, 95]. More recently, it was suggested that the presence of an effective antiviral inflammatory response in the presence of adequate immune regulation could be associated with protection during dengue secondary infection [96]. However, as highlighted in a recent review on dengue pathogenesis, it is worth noting that other infectious diseases and inflammatory disorders result in elevated cytokines without the attendant increased vascular permeability seen in severe dengue [97]. Indeed, one of the major challenges in dengue, magnified due to absence of good animal models of disease, is to dissect those elements of the host immune response that are causally linked to capillary permeability from those that simply reflect the normal host immune response to a pathogen [97].

Independent of this, antibodies specific for the NS1 viral protein may form immune complexes with the NS1 protein in the circulation and on the surface of infected cells leading to complement activation [98]. An additional risk factor for DHF is believed to be dependent on autoimmune responses against cross-reactive viral components. For example, antibodies specific for dengue virus NS1 protein may induce platelet lysis and/or nitric oxide-mediated apoptosis of endothelial cells, contributing to thrombocytopenia and vascular damage [99–104]. 

The serious complications observed during dengue infection occur as plasma viremia is resolving. This is thought to be immunologically mediated. Tam et al. performed a randomised trial to verify the effects of short-course oral corticosteroid therapy in early dengue infection. No association between treatment allocation and any of the predefined clinical, hematological, or virological endpoints was found [105]. Unexpectedly, the steroid doses administered were not immunosuppressive. Based on these observations it was suggested that rather than dengue-mediated vascular permeability being T-cell mediated, an alternative pathogenetic mechanism could involve the dengue soluble complement fixing antigen or the viral NS1 protein. Indeed, it was recently proposed that during the late stages of clinically apparent dengue infection, secreted DENV NS1 protein may bind to prothrombin and inhibit its activation, which in turn could contribute to the prolongation of activated partial thromboplastin time and haemorrhage in DHF patients [106]. Also, previous studies on the virological course of dengue infections in monkeys have shown that the peak of cellular infection occurs at the end of the viremic phase. Accordingly, it was proposed that dengue vascular permeability syndrome could be the equivalent of a viral toxicosis induced by circulating NS1 protein [107]. 

Whilst higher levels of viremia and circulating NS1 protein have been associated with dengue disease severity [108], collective results arising from different epidemiological settings are inconclusive, showing variations depending on the infecting serotype and patient immune status [109–112]. Therefore, the usefulness of these markers for the recognition of patients with increased risk of progression towards the more severe forms of dengue is still limited.

### 2.4. Underlying Basis for the Emergence of DHF in Particular Epidemiological Settings

Taking into account many of the factors described earlier and based on observations made during the 1981 Cuban epidemic of DF/DHF, Kouri and coworkers presented an integral hypothesis in which the association of different factors, such as immunological status, genetic background, host condition, viral strain, and epidemiological and ecological conditions, determines whether or not and to what extent, DHF will be involved in any particular epidemic [113]. Research conducted during the past 20 years strongly supports this unifying view of the situation [3, 39, 48, 114].

With the exception of Chile, Uruguay, and Cuba, that experience occasional epidemics, resulting from introduced virus, DF is endemic in Latin America and the Caribbean region. Cuba is a relatively small island with a well-integrated medical health and research infrastructure. When combined with the epidemiological history of DF in Cuba, this situation has provided a unique opportunity to investigate the specific risk factors for severe illness in detail [48]. Firstly, it is important to realise that from the end of World War II until 1977, dengue virus was not evident in Cuba. This was supported by a national seroepidemiological survey in 1975, which identified only 2.6% of the population with DENV hemagglutination-inhibition (HI) antibodies. Importantly, most of the positives were individuals older than 45 years [115, 116]. However, in 1977, based on serological evidence [117] it was estimated that up to 44.5% of all Cubans became infected by an introduced Asian strain of DENV-1. Nevertheless, no cases of DHF were recorded. These results demonstrate that in the absence of heterologous immunity, primary DENV-1 infections did not result in cases of DHF and, bearing in mind that subsequent DHF epidemics in Cuba all involved secondary infections (see later), the results strongly support the contention that secondary infections by heterologous serotypes are a very important risk factor for DHF as proposed previously [118].

During the past 28 years, three DENV-epidemics involving DHF have occurred in Cuba. The first epidemic started in 1981 [113], the second was seen in 1997 [65], and the third occurred in 2001 [63]. During each epidemic, secondary infection was demonstrated as the most important host risk factor for DHF. Additionally, specific sequential virus serotypes were associated with severe disease, independent of the time-gap between the primary and secondary infection. For example, in Cuba, two epidemics of DHF have been associated with primary infections due to an Asian strain of DENV-1 and secondary infections due to DENV-2 (i.e., DENV-1/DENV-2) [119, 120]. Significantly, during the 1997 Cuban epidemic, it was demonstrated for the first time that there was a higher risk of DHF in DENV-1/DENV-2 individuals when the average time gap between primary and secondary infections was about 20 years as opposed to 4 years, a more commonly reported timespan [64]. Moreover, comparison of attack rates and case fatality rates, in the same age groups, revealed that during the 1997 epidemic the rate in patients older than 15 years of age was 40 times higher than during the 1981 epidemic [121]. 

Subsequently, another Cuban epidemic, caused by DENV-3 and involving cases of DHF, occurred in 2001. Thus, DHF occurred in DENV-3-infected Cubans 24 years after primary exposure to DENV-1 infection [122]. Interestingly, Cubans infected sequentially with DENV-1/DENV-3 were associated with severe disease whilst those infected sequentially with DENV-2/DENV-3 were associated with milder disease or asymptomatic infections [63, 123]. Additionally, the DENV-3 immune individuals, infected during the 2001 Cuban epidemic, revealed differences in the neutralization capability of their sera to different DENV-3 strains belonging to different genotypes [124]. This observation might be anticipated taking into account that differences in neutralisation capability have been found using different genotypes of DENV-2. However, it was highly significant to find differences in neutralisation against strains belonging to the same genotype [125]. Moreover, the strains involved in the Cuban 1981, 1997, and 2001 epidemics [80, 125, 126] had previously been associated with severe epidemics and therefore had the potential to produce DHF. Nevertheless, in all of these epidemics, an extremely high number of primarily infected individuals were asymptomatic [64]. 

Using human volunteers, Dr. Albert Sabin was the first scientist to demonstrate that heterotypic immunity can prevent disease induced by a different dengue virus serotype [127]. Whilst DENV-1 immunity did not appear to prevent DENV-2 infections, partial immunity may have downregulated infections, thus reducing severity to mild disease during secondary dengue infections. It has been postulated that if virological factors are involved in determining disease severity, they may reflect common antigenic determinants shared between the first and second infecting viruses [39]. An exceptional illustration of this phenomenon is the neutralization of American genotype DENV-2 by human antibodies to DENV-1 [81]. These results suggest that the apparent lower virulence of American genotype DENV-2 results from a DENV-1 like surface epitope on the DENV-2 that permits partial neutralization (and downregulation of disease) by DENV-1 antibodies [81]. In contrast, Asian genotypes DENV-2 are poorly neutralised by human antibodies to DENV-1 [128]. Furthermore, a significant increase in the mean titre of homologous DENV-1 neutralizing antibodies and a significant decrease in heterologous antibodies to DENV-2 American genotype were reported in a long-term study in Cuba [128]. This finding may reflect time-dependent changes in severity of disease observed following secondary dengue infection.

On the other hand, case fatality rates were observed to increase month by month during epidemics that were studied in Cuba. Taking into account that DENV-2 epidemics occurred in 1981 and 1997, Guzmán and coworkers proposed a neutralisation escape mutant hypothesis based on the association of severe disease with dengue secondary infection [129]. Furthermore, during the DENV-3 epidemic that occurred in Havana in 2001, the same sequential increase in case fatality rates was observed [48, 85, 122].

 Although specific viral factors alone probably do not determine the severity of dengue infection in individual cases, the demonstrated increasing severity of infection with time during a single epidemic strongly argues that significant changes occur in the virus causing the epidemic. Indeed, host factors do not appear to explain this observation of increasing severity with time, because it is not logical to assume that the most susceptible individuals would all be infected towards the end of the epidemic. 

 The 1997 Cuban epidemic was the most severe reported in Cuba to date. Nevertheless, a search for evidence of the appearance of neutralisation-escape mutants proved negative. The structural gene sequences were highly conserved in viruses isolated at different times during the epidemic [126]. However, nucleotide substitutions were found in the nonstructural genes and in general they correlated with the time of sampling, showing a clear pattern of virus evolution during the epidemic [130]. Therefore, at least in this study, antibody-driven selection of escape mutants in the structural genes was not the key selective force. On the other hand, cytotoxic T-lymphocytes (CTLs) play a crucial role in controlling infection in RNA viruses, including dengue viruses [62, 131]. Variation in the epitopes recognized by CTL is common and frequently offers potential escape routes for mutant virus. Forthcoming studies will assess whether or not the reported mutations in NS1 and NS5 proteins [130] are represented in antibody inducing or CTL epitopes.

Regardless of which mechanism, that is, natural selection or genetic drift, is operating, it is likely that a fitter virus could be selected during the period of high transmission in individuals that have experienced secondary infections. However, it is a very difficult task to study dengue epidemiology because it is not only endemic in most tropical countries but there are four serotypes and many different genotypes often cocirculating. Nevertheless, Cuba represents a unique epidemiological setting for this kind of research because epidemics caused by only one serotype have occurred providing the opportunity for carefully defined epidemiological studies. 

Mutations in the nonstructural genes of DENV-2, isolated during the Santiago de Cuba epidemic, may correlate with increased efficiency of virus replication. Variation in nonstructural proteins has been also associated with increasing severity in epidemiological settings corresponding to endemic/epidemic transmission [132–136]. However, the specific relevance of these types of mutation has not yet been investigated thoroughly. This is in large part due to the lack of suitable animal models with which to study dengue virus “virulence” [51, 137]. 

During the most recent and severe DENV-4 epidemic in Puerto Rico in 1998, viruses were distinguished by three amino acid replacements in the NS2A protein (I14V, V54T, and P101T), which were fixed more rapidly than would have been expected by drift alone. This study demonstrates the significance of viral genetic turnover within a focal population and the potential importance of adaptive evolution during viral epidemic expansion [132]. In contrast, a retrospective phylogenetic study of events on the Southern Pacific islands three decades ago, where severe dengue was described in patients infected with the DENV-2 American genotype, recorded attenuation of this virus following a series of outbreaks involving nonsynonymous mutations, also in the NS2A gene [138]. 

Similarly, study of the population structure of dengue viruses transmitted in Aragua, Venezuela, during the period 2006-2007, under hyperendemic conditions also suggested that the nonstructural proteins could play an important role in DENV evolution. According to this particular epidemiological setting, changes in NS1, NS2A, and NS4B proteins were either favourable or adverse in terms of viral fitness. The authors argued that specific mutations could be associated with severe disease but some could be associated with mild disease due to the appearance of naturally attenuated strains [139]. 

The flavivirus NS2A protein is a small, hydrophobic, multifunctional membrane-associated protein involved in RNA replication [140, 141], host-antiviral interferon response [142–145], and assembly/secretion of virus particles [146–148]. In addition, the NS2A and the NS4B proteins may participate in the modulation of vector competence [149]. According to previous reports, changes in NS1 and NS4 proteins could be involved in viral attenuation [150, 151]. On the contrary, recent studies have demonstrated that mutations in the NS4B protein may increase the efficiency of DENV replication. In addition, it has been suggested that mutations in this protein may also be involved in species tropism of DENV and may even modulate the balance of efficient replication in mosquito and mammalian cells [152]. Moreover, it has been shown that a single amino acid in the nonstructural NS4B protein namely, L52F, confers virulence on DENV-2 in AG129 mice through enhancement of viral RNA synthesis [153]. 

Whilst these results suggest a possible role for the NS genes in determining viral fitness, the importance of the structural genes should not be overlooked. The sequences compared in the cited studies represent a consensus of those observed within each patient and may not necessarily represent the dominant variant present in the original clinical sample. For example, virus isolation using mosquito cell lines [154] is known to perturb the distribution of variants in the original clinical sample. This is particularly important given that studies of dengue virus populations sampled from individual humans or mosquitoes have revealed significant sequence variation [155]. Consequently, a greater focus on studies of viral population variation during epidemics is needed and the data should be obtained directly from clinical samples. 

The demonstration of long-term transmission of defective dengue virus in humans and mosquitoes has added a new dimension to the study of dengue evolution. The increased frequency of the “stop-codon” strain was concomitant with a major reduction in DENV-1 prevalence in Myanmar. The authors suggested that complementation between defective variants might provide a mechanism for the survival of “hyperparasites,” and this process of viral complementation could impact on pathogen transmission and virulence [156].

Obviously, more comprehensive approaches, including sequencing of larger numbers of viral genomes obtained directly from diverse clinical samples corresponding to longitudinal studies, are needed to examine how the genetic structure of dengue virus is influenced by heterotypic antibodies. 

Data obtained in two carefully planned clinical studies of dengue in Nicaragua demonstrate that the complex interplay between viral genetics and serotype-specific immunity determines the risk of severe dengue disease. Indeed, these data provide insights into viral evolution and the interaction between viral and immunological determinants of fitness and virulence. The abrupt increase in disease severity across several epidemic seasons of DENV-2 transmission coincided with clade replacement events. Interestingly, DENV-2 strains corresponding to NI-1 clade caused severe disease specifically in children who were immune to DENV-1, whereas DENV-3 immunity was associated with more severe disease among NI-2B infections, signifying that mutations altering the neutralization profile of some DENV strains can lead to increased viral fitness [157]. 

 Most dengue virus genomic studies have been directed at identifying the origin and genetic relatedness of the viruses causing epidemics. Other studies have focussed on identifying genetic markers associated with severe disease and comparing viruses isolated from DF and DHF/DSS cases within the same epidemiological setting. However, genetic variations have not been consistently associated with differences in clinical outcome. Conversely, introduction of new genotypes/serotypes with replacement/displacement of the existing viruses or changes in viral populations during an interepidemic period, extinction events, and sustained transmission of dengue virus due to repeated introductions have all been related to changes in the severity pattern of local epidemics [134, 158–161]. 

Recently, in Vietnam, the introduction of the Asian 1 genotype of DENV-2 led to the complete replacement of the resident Asian/American genotype of DENV-2. The transmission fitness advantage of Asian 1 viruses was attributed to this virus attaining higher viremia levels in humans [162]. However, there are multiple factors implicated in the transmission dynamic of DENVs that remain unclear. Epidemiological data have suggested that fitness is always context dependent and that as the immunological landscape changes, viral lineages that evade cross-immunity will be at a selective advantage [163]. 

 Clearly, there is still wide scope for research on the molecular basis of dengue virus epidemiology and pathogenesis. We need to know whether or not a circulating dengue virus that produces an asymptomatic infection in one host differs in sequence from the same virus that causes a fatal infection in another host. We also need to know if different tissues [164] within a single host house the same dominant dengue virus strain. Similarly, does the virus that circulates in a single epidemic have the same sequence in individuals with different histories of dengue infection?

### 2.5. Intrahost Genetic Variation

The population genetics and evolutionary epidemiology of RNA viruses have been reviewed [165]. The authors describe migration or gene flow as a factor to consider during RNA virus evolution. Accordingly, they advocate that migration must not only be understood at a macroscopic level (i.e., among hosts within a population, among populations, or between host species), but also within a single infected individual. From the site of inoculation, viruses can be transported to several tissues, generating intrahost spatial variation. This has been studied in Hepatitis C virus, family* Flaviviridae* [166]. However, the effect of a nonhomogeneous population distribution on the spread, fitness, and variability of virus populations has not been studied extensively. Nevertheless, a positive correlation between migration rate and average fitness of the population has been observed [167]. The outcome of acute Hepatitis C infection has been attributed to the evolution of viral quasispecies [168]. Large-scale sequencing of complete viral RNA genomes obtained directly from clinical samples is needed to investigate the role of variation in viral populations on dengue pathogenesis. The purifying selection that phylogenies have revealed [169] thus far may be misleading because most of the sequences analysed over the years were obtained from tissue culture viral isolates. Additionally, an intrinsic inadequacy of utilising consensus sequences to make inferences concerning the fitness of viral populations is that consensus sequences only reflect the majority nucleotide at any given position of the viral genome. Consequently, low frequency variants will remain undetected.

Examination of the viral population structure in mosquitoes and patients has revealed that the sequences of the major variants are the same but the extent of sequence variation seen with the mosquitoes is generally lower than that seen with the patients, suggesting that the mosquito contributes to the evolutionary conservation of dengue virus by maintaining a more homogenous viral population and a dominant variant during transmission [170]. In addition, by studying the evolutionary relationships of DENV-1 viruses that have circulated in French Polynesia and the viral intrahost genetic diversity according to clinical presentation, Descloux and coworkers suggested for the first time that clinical outcome may correlate with intrahost genetic diversity [171]. On the other hand, a recent study in Vietnam showed no relationship between the extent and pattern of DENV-1 genetic diversity and disease severity, immune status, or level of viremia. Interestingly, despite the high sequence conservation observed, clear evidence for mixed infection with the presence of multiple phylogenetically distinct lineages present within the same host was demonstrated [172]. 

Indeed, most attempts to investigate intrahost genetic variation in DENV characterised only a few viral genes or a limited number of full-length genomes. A new study in Nicaragua using a whole-genome amplification approach coupled with deep sequencing to capture intrahost diversity across the entire coding region of DENV-2 showed significant genetic diversity among genes [173]. However, the extension of that diversity was less than expected, suggesting strong purifying selection across transmission events as have been proposed previously [174–176]. 

Another point of view is that there is no reason to ignore vector-driven selection [177]. However, the interaction between virus and vector has been less extensively explored. By comparing the ability of DENV-1 isolates from Thailand, spanning a 24-year period, to infect and be transmitted by *St. aegypti*, Lambrechts et al. found that a major clade replacement event in the mid-1990s was associated with a higher transmission potential of the isolates belonging to the new clade. Higher transmissibility was mainly due to a higher infectious titre of virus in the vector's  haemocoel, which is predicted to result in a higher probability of transmission. This finding supports the hypothesis that major clade replacement events can be driven by natural selection and emphasizes the potentially important role of vector-virus interactions in DENV evolution [178]. 

Since the 1900s, the extrinsic incubation period (EIP), that is the time taken for the viremic bloodmeal to be amplified in the mosquito and then transmitted to a new host, had been recognized as an important component of DENV transmission dynamics. The DENV EIP is generally considered to be between 8 and 12 days [17]. Nonetheless, different factors can induce variations in EIP. For example, considerable degrees of variation in EIP have been shown to vary depending on the specific DENV strain studied. Mosquitoes feeding on humans infected with an unadapted strain of DENV-1 had shorter EIPs (14 days) than mosquitoes feeding on humans infected with strains at low mouse passage levels, where the EIP was 22 days [127]. In addition, long EIPs have been observed with dengue virus attenuated strains [179, 180]. Likewise, highly controlled laboratory studies have demonstrated the effect of distinct genotypes, serotypes, and mosquito population on the EIP [181, 182]. Taking into account the advanced technologies available in molecular biology, new avenues for the study of virus-vector interactions should reveal new mechanisms involved in dengue virus transmission dynamics.

## 3. Disease Control Strategies

Increasing human and associated mosquito population densities and mobility of humans and commercial goods are the main factors that have determined the very successful reemergence of DF and DHF during recent decades. In contrast with YFV, which exploits the same mosquito species (*St. aegypti*) to infect humans, the dengue viruses have evolved to become independent of the need for a reservoir sylvatic environment with which to sustain their epidemicity. Thus, in the absence of effective control strategies we are faced with the prospect of further increases in morbidity and mortality due to the dengue viruses. Currently, several different approaches (reviewed after) are being developed in the future hope of alleviating this “scourge” of modern times.

### 3.1. Vector Control

History has shown that vector control measures can be effective in reducing arthropod-borne virus diseases [120, 183–186]. However, many developing countries do not have the necessary resources and infrastructure for successful eradication measures to be implemented and sustainable in the manner that has been achieved in Singapore and Cuba [187]. This situation is exacerbated by the emergence of resistance to insecticides and the environmental issues arising from the use of potentially toxic chemicals [188]. Today, dissemination of insecticide resistance throughout vector populations is much faster than the rate of development of new insecticides. In addition, the existence of cross-resistance, based on the activation of general detoxifying mechanisms in the vector, can shorten the useful lifespan of alternative insecticides or even prevent their implementation [189]. 

New approaches to vector eradication including the use of naturally occurring plant insecticides/larvicides [190] and vaccines that induce antibodies to impair vital functions in mosquitoes [191] are being considered but such approaches are unlikely to provide effective vector control measures in the near future. 

Primary prevention of dengue is largely dependent on larval and adult mosquito control. *St. aegypti* surveillance has relied heavily upon larval indices. However, this has been strongly criticised as they provide little information to determine the risk of DENV transmission. The studies of Bisset Lazcano and colleagues used pupal surveillance for the *St. aegypti *control programme in Cuba and focused on the most productive mosquito water containers [192]. In urban areas, *St. aegypti* breed on water that collects in artificial containers such as plastic cups, used tyres, broken bottles, flowerpots, and other water traps. Elimination of these containers is the most effective way of reducing the mosquito breeding grounds. The use of insect repellents, mosquito traps, and mosquito nets in the home can also be moderately effective in reducing the number of bites due to mosquitoes.

Novel alternative approaches have also been investigated. In Vietnam, trials were conducted in which children and local communities were encouraged to place, a known mosquito predator, the crustacean *Mesocyclops* [193], in water tanks and discarded containers where *St. aegypti* are known to thrive [194]. The concept exploited the principle that this procedure might be more cost-effective and environmentally friendly than the use of pesticides. Over a period of years and in defined rural provinces of Viet Nam a reduction in mosquitoes and dengue fever was observed [195, 196]. However, such approaches are only likely to be successful in regions of countries with the organisational infrastructure and appropriate community attitudes. A community education strategy is utilised to promote participation in dengue prevention in Cuba, resulting in reduced mosquito vector infestation levels. The main principle has been to increase community participation in decision-making and strengthening the competence of the medical teams and community working groups [197]. Whether or not a similar approach could be successfully applied to major cities and urban areas in other countries remains to be seen. 

An alternative approach involves infecting *St. aegypti* with the bacterium Wolbachia [189]. Early studies suggest that this reduces the adult lifespan of the mosquito by 50% [198]. This is important because the adult female mosquito is the primary vector of the virus. Insects infected by Wolbachia transmit them transovarially to the next generation. Thus, by reducing the lifespan of the mosquito, virus-vector competence, and virus transmission efficiency, a significant reduction of *St. aegypti *should be observed. Another important feature of Wolbachia is its ability to induce resistance to a variety of pathogens, including DENV, in its insect hosts [199]. In the transinfected *St. aegypti*, all the three different types of Wolbachia, wAlbB, wMelPop-CLA, and wMel, induce significant inhibition of DENV replication and dissemination, resulting in either complete or partial block of virus transmission [200–202]. Recent studies also show that Wolbachia induces production of reactive-oxygen species which then activate the Toll-pathway to induce expression of antiviral effectors [203]. Interestingly, it was recently demonstrated that native Wolbachia symbionts limit transmission of DENV in *St. albopictus* by restricting the delivery of infectious viral particles from the mosquito saliva when biting. These results might therefore explain the low vector competence of *St. albopictus* for dengue and thus its relatively weak contribution as an epidemic dengue vector [204]. 

Understanding how Wolbachia density is regulated by mosquito hosts and how the Wolbachia machinery controls its replication will facilitate the current effort to eliminate dengue through Wolbachia-based population replacement [205].

The genetic structure of *St. aegypti *populations and its implications for potential mosquito releases have been studied in Queensland, Australia [206], and Tri Nguyen village, Vietnam [207, 208]. Populations of *St*. *aegypti* artificially infected with strains of *Wolbachia pipientis* that interfere with its vector competence are being backcrossed into wild mosquito genetic backgrounds from north Queensland and assessed as potential candidates for release [209]. In addition, a pilot release—http://www.eliminatedengue.com/project/vietnem/progress—of infected mosquitoes has been authorised to take place from April 2013 on Tri Nguyen village (611 households) on Hon Mieu Island in central Vietnam. Subject to satisfactory results larger scale studies could be launched within five years [210].

Promisingly, studies related with the effect of Wolbachia on insecticide susceptibility in lines of *St. aegypti* have demonstrated that spreading Wolbachia infections are unlikely to affect the efficacy of traditional chemical methods of controlling mosquito outbreaks [211].

### 3.2. Development of Vaccines against Dengue Viruses

 It is generally agreed that vaccination can provide an effective method with which to control virus diseases. In the case of the dengue viruses, the four serotypes are sufficiently antigenically different that it is considered necessary to produce four monovalent vaccines, which will then be mixed to produce a tetravalent immunological response. This is a logical approach that has previously been employed successfully with the three monovalent poliovirus vaccines. However, as discussed earlier, the dengue viruses also present the problem of antibody mediated enhancement of disease severity [75, 118, 212]. Long-term protection is essential as severe dengue has been observed in individuals secondarily infected more than 20 years after the primary infection [63, 85]. As it is virtually impossible to test whether or not the tetravalent formula would overcome this potential problem, an element of uncertainty might prevail following the introduction of the vaccines in Asia and/or Latin America where dengue viruses are most prevalent. In addition, although the protective role of neutralizing antibodies is recognised, correlates of protection need to be defined [213]. 

Several different approaches are being employed to develop dengue virus vaccines. The vaccine pipeline includes live empirically attenuated vaccines, newer live attenuated vaccines developed using infectious clone technology, genetic vaccines using virus and plasmid vectors, and many recombinant subunit vaccine candidates [214].

 Potential vaccines already progressing through clinical trials include a live attenuated tetravalent vaccine, produced via serial subculture in primary dog kidney cells [215–217]. Another approach involves the use of genetic modification of dengue viruses to attenuate their virulence [218–222]. Although vaccine candidates based on infectious virus have shown the greatest progress amongst different dengue vaccine approaches, there are safety concerns associated with their use based on potential reactogenicity, interference amongst the viruses, possible reversion to native virus, and possible increase of virus infectivity and/or virulence via antibody dependent enhancement [223].

 An alternative approach is based on the use of the live attenuated YFV 17D vaccine as a backbone for the production of four chimaeric live attenuated viruses in which the prM and E genes of the 17D vaccine virus are replaced by the corresponding genes of the four dengue virus serotypes [224–226]. Preclinical studies demonstrated that the tetravalent vaccine is genetically and phenotypically stable, nonhepatotropic, less neurovirulent than the tried and tested YFV 17D vaccine, and does not infect mosquitoes by the oral route. Vaccine reactogenicity, viremia induction, and antibody responses have been investigated in phase 1 trials in the USA, the Philippines, and Mexico. Preclinical and clinical trials showed favourable immunogenicity and short-term safety of this vaccine [227]. Relatively favourable results of phase 2 trials were published recently. A surprising lack of efficacy against DENV2 was observed, and the fact that DENV2 was the prevalent serotype during the study diminished the overall vaccine efficacy in this setting [228].

 Several possible causes of this apparent failure have been proposed including significant genetic differences between the circulating DENV-2 genotype and the strain incorporated in the yellow fever chimaeric vaccine, imbalanced viraemias, or immune responses due to interference. Nevertheless, Sanofi's CYD dengue vaccine has been discussed as a potential “75% solution” referring to the vaccine's efficacy towards three of the four DENVs in the context of potential antibody-dependent enhancement. The general view seems to be that this approach would be inappropriate [229]. Probably, the most relevant issue is related to the long-term safety of such vaccines. DENV-2 has been associated with severe disease in several epidemiological settings. In fact, studies in Cuba demonstrated that disease severity increased notably when infection with DENV-2 follows infection with DENV-1 at an interval of 20 years [64], probably due to a significant decrease in mean titre of heterologous neutralizing antibodies [128]. Thus, there is justified concern that CYD vaccinated individuals could develop severe disease if infection with DENV-2 occurs after a relatively long interval of time. On the other hand, depending on the DENV-2 genotype that might subsequently circulate, it cannot be ruled out that heterologous neutralisation might lead to a satisfactory immune outcome, as occurred in Cuba during the 2001-2002 epidemic caused by DENV-3, where most DENV-2 immune individuals (infected in 1981) developed asymptomatic DENV-3 infections whilst a high proportion of the DENV-1 immune cases suffered overt disease [63, 230]. In summary our limited understanding of the underlying processes of the immunopathological response to primary and successive infections with the four DENV largely determines our inability to predict the clinical outcome [231, 232]. Nevertheless, ongoing large-scale phase 3 studies in more than 30,000 volunteers in ten countries in Latin America and Asia should provide critical data with which to overcome initial problems identified with the CYD dengue vaccine candidate.

A similar approach is being developed based on the attenuated DENV-2 virus, DENV-2 PDK-53, and three chimaeric viruses containing the prM and E genes of DENV-1, DENV-3, and DENV-4 virus in the genetic backbone of the DENV-2 PDK-53 virus (termed DENVax). Based on the safety, immunogenicity, and efficacy in preclinical studies in animal models, phase 1 clinical testing of tetravalent DENVax has been initiated [233–235]. This candidate might have advantages as the DENV backbone could reduce the risk of unanticipated effects due to the YFV NS proteins present in the Sanofi vaccine candidate [228].

Potential vaccines not yet progressing through clinical trials include the development of subunit vaccines based on domain III of the dengue virus envelope protein. Recombinant fusion proteins formed by domain III and P64k protein from Neisseria meningitides expressed in *E. coli* induce functional and protective immunity in mice and nonhuman primate models inducing highly serotype specific immune responses [236–238]. Additionally, the domains of each serotype have been engineered in tandem in a yeast expression system [239]. A recombinant adenovirus system has been utilised to express the DENV NS1 proteins [240]. The paediatric measles vaccine has been modified to express a fragment of the DENV-1 M protein together with domain III of the envelope protein [241]. More recently, the evaluation in mice of a novel domain III-capsid chimaeric protein expressed in *E. coli* provides additional evidence for a crucial role of cell-mediated immunity in protection against dengue virus [242–245]. Finally, a  novel single-dose lipidated consensus dengue virus envelope protein domain III (LcEDIII) subunit vaccine was shown to induce humoral and cellular immune responses in mice [246]. This group also evaluated the efficacy of the newly developed water-in-oil-in-water multiphase emulsion system, termed PELC plus CpG oligodeoxynucleotides, in potentiating the protective capacity of dengue-1 envelope protein domain III concluding that it could be a promising adjuvant for recombinant protein candidates [247].

 Whilst subunit vaccines may have some advantages in terms of type-specific neutralisation with a low potential for inducing ADE via cross-reactive antibodies and low reactogenicity, multiple doses are usually needed to ensure long-term immune protection. However, it has been suggested that recombinant domain III vaccine could function as a booster if used in combination with live vaccine [223].

It is anticipated that within the next five years, increased understanding of the basis for dengue pathogenesis [248] and the protective immune response to DENV will improve our ability to develop safer and more effective vaccines [223]. 

Critical issues in dengue vaccine development have been reviewed [249]. Of relevance is the potential impact of vaccination on the evolution of naturally occurring DENVs. Vaccination could ultimately produce an environment where relatively low transmission of natural DENV occurs. This is especially relevant if vaccination is focused on a selected portion of the population, thereby increasing stochastic events that will allow new DENV genotypes to emerge possibly with greater virulence. Furthermore, dengue vaccination may produce a background of low titres of enhancing antibody to specific DENV serotypes, resulting in the emergence of specific serotypes in a population. Recent studies suggest that strain diversity may limit the efficacy of monoclonal antibody therapy or tetravalent vaccines against DENV as neutralization potency generally correlates with a narrowed genotype specificity [250]. Consequently, a better understanding of dengue immunopathogenesis will assist not only development of therapeutic interventions but also the understanding of dengue vaccine efficacy or vaccine adverse events [97]. Therefore, laboratory surveillance of dengue needs to be improved considerably to increase our knowledge of the circulating viruses at the molecular level, preferably before the introduction of a vaccine on a large-scale.

### 3.3. Development of Antivirals against Dengue Viruses

Whilst no approved antiviral therapeutic agents are available to treat individuals presenting with symptoms of DF/DHF, several potential virus inhibitors are under consideration for further development. The dengue viruses provide a variety of potential targets for inhibitors of infection/replication. As Hepatitis C virus (HCV) is also a member of the *Flaviviridae*, antivirals under development to control disease due to HCV may also prove to be effective against the dengue viruses. One of the major problems likely to be encountered is drug resistance. Consequently, the discovery and development of at least two antivirals that attack different viral targets should be a minimum goal. Another major hurdle for dengue viruses is the lack of availability of a validated animal model that faithfully reflects DHF and DSS observed in patients. 

The NS5 viral RNA dependent RNA polymerase (RdRp) and the methyl transferase, as well as the NS3 protease and helicase, are considered good targets for inhibitors of dengue infection, because they are all major components of the replicative viral complex [251]. The viral envelope (E) protein is also a good target for antivirals. The use of E protein-specific monoclonal antibodies has been shown to have some potential in this context [252]. The NS1, prM, and capsid protein of the dengue viruses have not been studied at the same level of intensity, and thus there are few if any potential antivirals against these targets. 

One approach that appears quite successful both *in vitro *and *in vivo* has been the demonstration that specific antisense morpholino oligomers can inhibit dengue virus replication [253, 254]. However, major efforts are required to reduce the risk of toxicity and to provide safe and effective delivery systems for these oligomers [252, 255]. 

Other approaches are being utilised to identify flavivirus chemotherapeutic agents, including screening known inhibitors of other viruses, rational design based on protein crystal structures or secondary viral RNA structures, optimization of known viral inhibitors, use of humanized antibodies, use of immunoglobulins, and nucleic acid-based therapy [256].

Polyoxotungstates and sulphated polysaccharides show some potential as viral inhibitors. They impair flavivirus adsorption and entry into host cells *in vitro*, apparently by binding to the cell surface [257, 258]. Sulphated galactomannans protected mice from lethal YFV infection when inoculated simultaneously with the virus [259].

The licensed drug Ribavirin has been used to treat a number of RNA viral infections. It functions as an RNA cap analogue and mutagen, causing errors in synthetic pathways [260–262]. However, the *in vitro* and *in vivo *activity of Ribavirin against YFV and DENV was poor [263–265]. Prophylactic Ribavirin treatment of rhesus monkeys infected with DENV had little effect on viremia [266] and in mice, intraperitoneal administration of Ribavirin had no effect on survival following intracerebral inoculation with DENV. However, treatment with Ribavirin-2′,3′,5′-triacetate, a prodrug of Ribavirin, resulted in a significantly increased survival time and rate, possibly due to its higher ability to cross the blood-brain barrier [267]. 

Nucleoside analogues, characterized for chemotherapeutic use against HIV and Hepatitis B virus, show inhibitory activity in cell culture against YFV, DENV, and West Nile virus (WNV) [268]. Rather than blocking RNA replication, some analogues inhibit flaviviruses by inhibiting nucleoside triphosphate synthesis in host cells. For example, 6-azauridine acetate, pyrazofurin, and 2 thio-azauridine inhibit orotidine monophosphate decarboxylase (OMPDC). In contrast, mycophenolic acid and Ribavirin-2′,3′,5′-triacetate inhibit inosine monophosphate dehydrogenase (IMPDH) and block viral RNA synthesis [269, 270]. Carbamate prodrugs have also been recommended as IMPDH inhibitors since they show *in vivo *activity [271]. Recently a uracil-based multifunctional compound was shown to have strong activity against dengue virus. It is likely that the mechanism of action of the antiviral activity of this compound is through inhibition of the enzyme, IMPDH [272]. 

An alternative strategy in the search for effective antivirals that potentially reduces the lead-time for their development is to identify drugs already licensed for use to control diseases other than those caused by the target virus. For example, the aminoglycoside, Geneticin (G418), was recently shown to have antiviral activity against bovine viral diarrhea virus (BVDV). Since BVDV, DENV, and YFV all belong to the virus family *Flaviviridae*, it seems possible that a common step in their life cycle might be affected by this aminoglycoside. Geneticin prevented the cytopathic effect resulting from DENV-2 infection of BHK cells, in a dose-dependent manner [273]. However, Geneticin had no detectable effect on YFV in BHK cells. 

Ivermectin, a broadly used antihelmintic drug, displays specific inhibitory unwinding activity against helicases from several flaviviruses, including YFV, DENV, and WNV with the half maximal inhibitory concentration (IC_50_) values in the submicromolar range [274]. Preliminary studies indicate higher binding efficiency with YFV than with DENV. Nevertheless, disappointingly, Ivermectin did not protect hamsters against infection with YFV. Structure-based optimization may result in analogues exerting potent activity against flaviviruses both *in vitro *and *in vivo*.

Doxorubicin is an antineoplastic antibiotic obtained from *Streptomyces peucetius*. This antibiotic exhibits *in vitro *antiviral activity against the YFV17D vaccine strain and the DENV-2 NGC strain. Doxorubicin proved to be cytotoxic in uninfected host cells. However, a novel derivative of doxorubicin, SA-17, showed excellent antiviral activity against DENV and markedly reduced cytopathogenicity [275]. Dose-dependent anti-DENV activity was confirmed using a dengue reporter virus. Time-of-drug addition studies indicated that SA-17 acts at an early stage of the replication cycle. It does not inhibit the replication of the replicon and thus does not work at the level of the viral replication machinery. Further studies revealed that SA-17 exerts it activity *via *a virucidal effect, even when using very high titres of the virus as the inoculum. 

A large number of small molecules derived by computer modelling of known enzyme domains were screened for inhibitory activity against DENV-2 virus. Two of these molecules, ARDP0006 and ARDP0009, inhibited DENV-2 with high efficiency. ARDP0009 had no apparent toxicity at the concentrations tested. Selectivity indices calculated for ARDP0006 and ARDP0009 were comparable to those calculated for Ribavirin which has demonstrated inhibition of the DENV-2-O-methyltransferase NS5 [276] and HCV replication when used in combination with interferon. Antiviral activity, *in vitro,* of 3′,5′ di-*O*-trityluridine has also been identified. The compound inhibits DENV and YFV replication by targeting the elongation process of the viral NS5 RNA-dependent RNA polymerase. A nucleoside analogue (T-705) which is a substituted pyrazine compound that has been used in clinical trials for the treatment of human influenza virus infection is an analogue of T-1106, a known HCV polymerase inhibitor [277]. T-705 significantly improved survival and disease parameters in YFV-infected hamsters despite the lack of good *in vitro *antiviral activity. These studies highlight the possibility that nucleoside analogues could potentially be developed for flavivirus therapy although more potent compounds with reduced toxic effects on the host cells will need to be generated.

Flaviviral inhibitory activity has also been observed with plant extracts. Boesenbergia rotunda (L.) Mansf. Kulturpfl. (BR) is a common spice belonging to a member of the ginger family (Zingiberaceae). Some of the BR compounds, such as flavanoids and chalcones, have been shown to be pharmaceutically active. The chalcone, cardamonin, isolated from BR, was recently reported to exhibit appreciable anti-HIV-1 protease inhibition [278]. Moreover, inhibitory activity by six compounds isolated from BR has also been demonstrated on DENV-2 virus NS3 protease activity.

In conclusion, whilst mosquito control strategies have been shown to be successful in reducing the incidence of dengue infections, such methods are most effective in those tropical/subtropical countries that have well-developed human and environmental health infrastructures. Clearly, there is a real need for more effort to understand the complex epidemiology and pathogenesis of the dengue viruses to expedite the development of suitable vaccines and/or antiviral therapies. Although some vaccine candidates appear promising, as yet none has been licensed. Due to the presence of the four DENV serotypes, these viruses present a different situation from YFV, tick-borne encephalitis virus, and Japanese encephalitis virus. The question of whether or not deteriorating antibody levels will leave vaccinated people liable to the development of DHF via ADE will need to be addressed. Moreover, the relationships between the presence of neutralizing antibodies, the level of protection afforded and the duration of protection by each of the four serotypes will need to be critically assessed. There is a pressing need for global collective efforts to develop antiviral therapeutics with which to combat dengue viruses. The current trend of expanding our efforts on antiviral drug discovery is encouraging in this respect.
